# Synergistic Action of MCL-1 Inhibitor with BCL-2/BCL-XL or MAPK Pathway Inhibitors Enhances Acute Myeloid Leukemia Cell Apoptosis and Differentiation

**DOI:** 10.3390/ijms24087180

**Published:** 2023-04-13

**Authors:** Małgorzata Opydo, Anna Mlyczyńska, Ewa Mlyczyńska, Agnieszka Rak, Elzbieta Kolaczkowska

**Affiliations:** 1 Laboratory of Experimental Hematology, Institute of Zoology and Biomedical Research, Faculty of Biology, Jagiellonian University, 30-387 Krakow, Poland; 2 Laboratory of Physiology and Toxicology of Reproduction, Institute of Zoology and Biomedical Research, Faculty of Biology, Jagiellonian University, 30-387 Krakow, Poland; 3 Doctoral School of Exact and Natural Sciences, Jagiellonian University, 30-387 Krakow, Poland

**Keywords:** BCL-2 inhibitors, S63845, ABT-737, MAPK signaling pathway, apoptosis, differentiation, acute myeloid leukemia

## Abstract

Acute myeloid leukemia (AML) is a hematological malignancy characterized by excessive proliferation of abnormal myeloid precursors accompanied by a differentiation block and inhibition of apoptosis. Increased expression of an anti-apoptotic MCL-1 protein was shown to be critical for the sustained survival and expansion of AML cells. Therefore, herein, we examined the pro-apoptotic and pro-differentiating effects of S63845, a specific inhibitor of MCL-1, in a single-agent treatment and in combination with BCL-2/BCL-XL inhibitor, ABT-737, in two AML cell lines: HL-60 and ML-1. Additionally, we determined whether inhibition of the MAPK pathway had an impact on the sensitivity of AML cells to S63845. To assess AML cells’ apoptosis and differentiation, in vitro studies were performed using PrestoBlue assay, Coulter electrical impedance method, flow cytometry, light microscopy and Western blot techniques. S63845 caused a concentration-dependent decrease in the viability of HL-60 and ML-1 cells and increased the percentage of apoptotic cells. Combined treatment with S63845 and ABT-737 or MAPK pathway inhibitor enhanced apoptosis but also induced differentiation of tested cells, as well as altering the expression of the MCL-1 protein. Taken together, our data provide the rationale for further studies regarding the use of MCL-1 inhibitor in combination with other pro-survival protein inhibitors.

## 1. Introduction

The evasion of apoptosis, which leads to cancer initiation and progression as well as its resistance to treatment, is considered a hallmark of cancer [1]. Therefore, induction of apoptosis in cancer cells represents the principal goal of anticancer therapy. Both extrinsic and intrinsic apoptosis pathways can be activated in response to most anticancer strategies currently used, such as chemotherapy, radiotherapy, targeted therapy and immunotherapy [2]. In this matter, the knowledge of aberrant apoptotic machinery in cancer cells is of key importance to enhance the efficacy of therapy. As shown by numerous studies, one of the most common strategies that cancer cells employ to evade apoptosis is overexpression of pro-survival members of the BCL-2 protein family [3]. These proteins, namely BCL-2, BCL-XL, BCL-w, MCL-1 and BFL-1/A1, are essential regulators of the intrinsic apoptotic pathway. They function to counteract the activity of pro-apoptotic BCL-2 family members, such as BAX/BAK multidomain proteins and BH3-only proteins, thereby preventing mitochondrial outer membrane permeabilization (MOMP), which leads to caspase cascade activation and apoptosis [4].

Overexpression of anti-apoptotic BCL-2 family proteins was reported in various solid tumors and hematological malignancies [5]. Considering the latter, a number of studies have linked de-regulation of these anti-apoptotic proteins with pathogenesis and treatment failure in acute and chronic leukemias of myeloid origin [6,7,8,9]. Importantly, during myelopoiesis, several factors influence the survival, proliferation and differentiation of myeloid cells, in part by regulating the expression of BCL-2 family proteins. Members of the BCL-2 family function as critical nodes in complex regulatory networks, which make ultimate life/death decisions within a cell. Although BCL-2 family proteins do not have a direct role in myeloid cell proliferation and differentiation programs, they can either allow these programs to proceed or prevent them. Through such effects, the BCL-2 family proteins maintain cellular homeostasis [10]. Therefore, any alteration in the expression of its members disturbs the proper balance between cell death and cell survival signals and contributes to leukemogenesis [10].

Among leukemias, acute myeloid leukemia (AML) is the most common malignancy diagnosed in the adult population and accounts for about 80% of all cases [11]. It still remains a disease with poor clinical prognosis due to high incidence of relapses and low 5-year survival rate (40–50%) [11]. Intensive studies aimed to determine the molecular pathways of leukemogenesis recognized AML as a heterogenous disease, which results from a complex network of cytogenetic aberrations and molecular mutations [12,13]. As a result, in AML, excessive cell proliferation accompanied with a differentiation block and inhibition of apoptosis leads to the accumulation of abnormal myeloid precursor cells [13]. Moreover, the heterogeneity of AML is a key determinant of disease progression and poor treatment response. For the past 40 years, hematopoietic stem cell transplantation and chemotherapy based on anthracyclines and cytarabine remained the main treatments for AML. However, in 2017, there was a breakthrough, and new therapeutics were approved by the Food and Drug Administration (FDA) in the US, including targeted drugs, such as FLT3 inhibitor midostaurin, IDH2 inhibitor enasidenib and venetoclax, which belongs to the class of small-molecule BCL-2 inhibitors [14].

BCL-2 inhibitors, named BH3 mimetics, were designed to specifically block anti-apoptotic proteins from the BCL-2 family by binding to their hydrophobic groove and preventing their ability to inhibit pro-apoptotic family members. These agents can act as apoptosis inducers and/or sensitizers in cancer cells [15]. Thus far, a number of BH-3 mimetics with different specificities have been synthetized. ABT-737 and its orally available analog ABT-263 were one of the first BCL-2 inhibitors designed to bind with high affinity to BCL-2, BCL-XL and BCL-w [15]. ABT-737 induced apoptosis as a single agent in AML-derived cell lines in primary blasts and in AML stem cells and was effective at reducing the leukemia burden in vivo in a xenograft model of AML [16]. Moreover, BH3 profiling demonstrated myeloblast sensitivity to ABT-737 and its dependence on BCL-2 in the mitochondria [17], which prompted the development of a highly specific inhibitor of BCL-2 protein, ABT-199 (venetoclax). This BH3 mimetic induced apoptosis in multiple AML cell lines at nanomolar concentrations and inhibited leukemia progression in an in vivo murine AML xenograft model [18]. However, consequent studies revealed that cancer cells can acquire resistance to ABT-737 and ABT-199, which is partially mediated by the up-regulation of MCL-1, another member of the BCL-2 family. It was shown that high MCL-1 expression and BCL-2 phosphorylation markedly reduced the ability of ABT-737 to induce apoptosis [16], whereas up-regulation of MCL-1 and BCL-XL was found to be responsible for the resistance of myeloid leukemia cell lines to ABT-199 [19]. The increased MCL-1 protein expression was found to occur through transcriptional or post-transcriptional mechanisms [20,21]. Given the latter, MCL-1 can be phosphorylated at Thr163 by the ERK1/2 protein, thus increasing its stability and enhancing its anti-apoptotic activity [22]. Importantly, in addition to regulating the activity of anti-apoptotic proteins from the BCL-2 family, ERK/MAPK signaling pathway can provide an anti-apoptotic effect by up-regulating the expression of these proteins [21,22].

As MCL-1 is critical for the sustained survival and expansion of mouse as well as human AML cells [20], targeting the intrinsic apoptotic pathway through MCL-1 antagonism is a rational strategy to restore apoptosis in AML cells. Recently, a new specific small-molecule inhibitor of MCL-1, namely S63845, has been synthetized [23]. This agent has been shown to inhibit MCL-1 with a Ki < 1.2 nM and Kd of 0.19 nM with no concurrent binding to either BCL-2 or BCL-XL (Ki > 10,000 nM) [24]. Its pro-apoptotic activity has been observed in a number of hematological malignancies, including multiple myeloma, lymphoma and AML-derived cell lines in vitro and in vivo [24]. S63845 selectively killed cancer cells, which relied on MCL-1 for survival, by activating the BAX/BAK-dependent mitochondrial apoptotic pathway, confirming its on-target effects [23]. Moreover, the high efficacy of the combination of S63845 with other anticancer drugs, such as tyrosine kinase inhibitors, cytarabine or docetaxel, was shown in hematological malignances as well as in solid tumors [23,25,26]. Recently, MIK665 (S64315) derived from S63845 has been introduced to clinical trials for hematological malignancies (NCT04629443, NCT02979366, NCT02992483, NCT04702425).

Considering all the above data, in this study, the antileukemic effects of an MCL-1 inhibitor, S63845, were examined in human AML cell lines when used as a single agent or in combination with BCL-2/BCL-XL inhibitor, ABT-737. Two AML cell lines, which differ with respect to their maturation level, were used in the study: HL-60 acute myeloblastic leukemia with maturation (FAB-M2) and human myeloblastic leukemia ML-1 (FAB-M5). These AML cell lines have been accepted as model systems for hematopoietic cell proliferation, differentiation and death studies. Given that AML is a disease associated with impaired cell differentiation and apoptosis, the impact of S63845 and ABT-737 on the induction of these processes was determined. Furthermore, as the expression of the MCL-1 protein is regulated by MAPK/ERK signaling, we also investigated the effects of MAPK pathway inhibition on the sensitivity of AML cells to S63845. Our in vitro data show that S63845 exerts cytotoxic effects on leukemic cells, and its anti-AML efficacy is enhanced by simultaneous targeting of BCL2/BCL-XL. Moreover, we demonstrate that the sensitivity of AML cells to S63845 can be increased by the inhibition of MAPK signaling pathway. Importantly, in the present study, we show for the first time that a combined application of S63845 and ABT-737 or MAPK inhibitor promotes not only apoptosis but also differentiation of human AML HL-60 and ML-1 cells.

## 2. Results

### 2.1. S63845 Exerts Cytotoxic Effect on AML Cells and Synergizes with ABT-737 in Reducing Leukemic Cell Viability and Count

**Figure 1:**
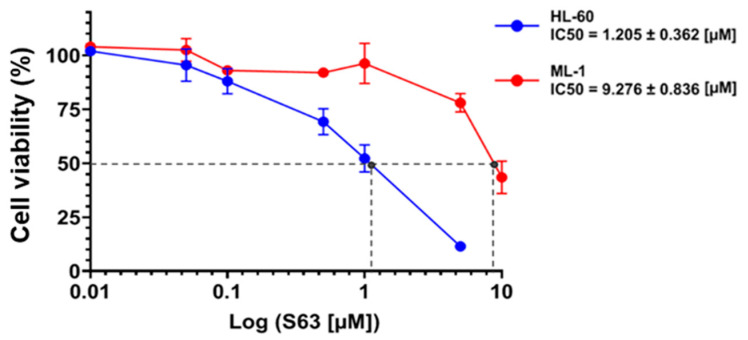
Dose–response curves of AML cell lines treated with MCL-1 inhibitor S63845 (S63) for 48 h. Analysis of HL-60 and ML-1 cell viability was performed using PrestoBlue assay. Based on the obtained data of three independent experiments performed in triplicate, the IC50 values for both AML cell lines were calculated.

The impact of S63845 on leukemic cell viability was assessed 48 h after application of the tested agent using the PrestoBlue assay. As shown in Figure 1, the exposure of AML cells to S63845 decreased their viability and was cell line dependent. HL-60 cells appeared to be more sensitive toward the agent, with the IC50 value being almost 7 times lower than that determined for ML-1 cells.

**Figure 2:**
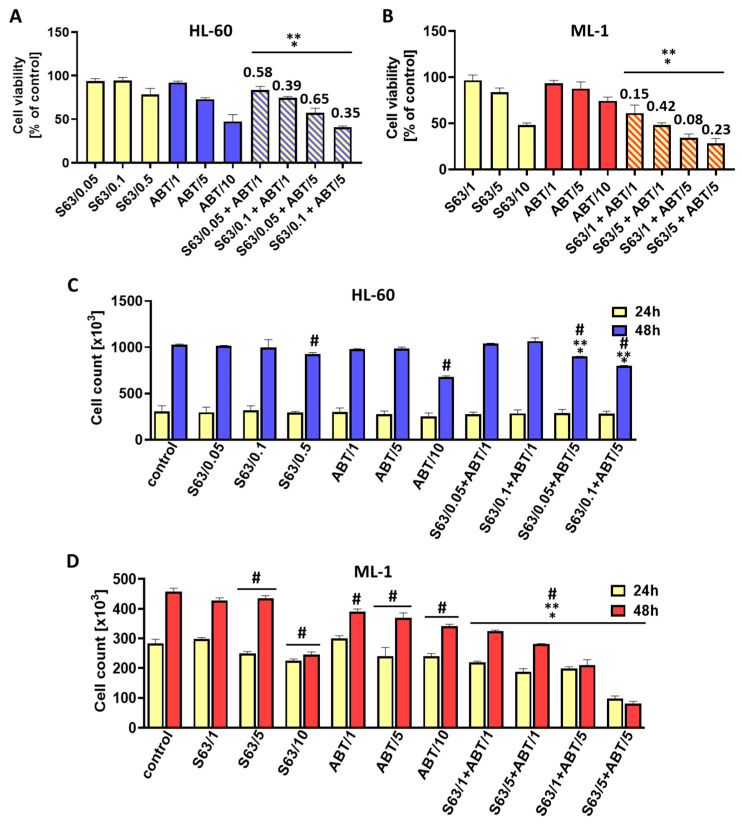
Effects of S63845 (S63) and ABT-737 (ABT) given as single agent or in combination on the viability and count of AML cells. Using PrestoBlue assay, the cell viability of HL-60 (**A**) and ML-1 (**B**) cells was determined at 48 h. The synergism between the tested agents was determined by the combination index (CI) analysis at a non-constant ratio. The CI values, generated using the CompuSyn software according to the Chou–Talalay method, are given in graph A and B. Using the Coulter method, the HL-60 (**C**) and ML-1 (**D**) cell count analyses were performed at 24 h and 48 h after application of the tested agents. Designations 0.05, 0.1, 0.5, 1, 5 and 10 indicate the agent concentrations (µM). Three independent experiments were performed in triplicate (**A**,**B**) or duplicate (**C**,**D**). Values are reported as the means ± SD. Values significantly different (*p* ˂ 0.05) according to one-way ANOVA are designated by * and ** compared to, respectively, S63845 or ABT-737—single treated group; # compared to the corresponding control.

Next, in order to examine the combined effects of S63845 and ABT-737 on AML cell viability, the concentrations of S63845 and ABT-737 below the IC50 values for each leukemic cell line were used. The concentrations of ABT-737 were chosen based on our previous studies, where we showed that ML-1 cells are more resistant to this BCL-2 inhibitor than HL-60 cells [27]. The exposure of both HL-60 and ML-1 cells to S63845 in combination with ABT-737 resulted in a decrease in cell viability to a higher degree than did each of the tested agents alone (Figure 2A,B, respectively). Importantly, although all combinations of the two inhibitors used to treat HL-60 cells significantly reduced their viability in comparison with the corresponding single-agent treatments, a decrease in HL-60 cell viability below 50% was observed only when S63845 and ABT-737 were used in the highest concentration of 0.1 µM and 5 µM, respectively (Figure 2A). In the case of ML-1 cells, all combinations applied caused a decrease in cell viability below 50% (Figure 2B). To confirm that the interactions between the tested agents were synergistic in HL-60 and ML-1 cells, the combination index values were calculated. In both leukemia cell lines, the CI values were below 1, indicating synergism. The CI values in HL-60 cells ranged from 0.35 to 0.65 and from 0.08 to 0.42 in ML-1 cells (Figure 2A,B, Supplementary Figure S1).

The cytotoxicity of S63845 and ABT-737 against AML cells was further explored by Coulter counter measurements indicative of cell number. A concentration-dependent decrease in leukemia cell count was observed 24 h and 48 h after application of the BCL-2 inhibitors alone (Figure 2C,D). However, the combination of S63845 and ABT-737 resulted in a further reduction in cell count as compared to the values obtained with each of these compounds alone. Additionally, the method revealed that the combination of the two BCL-2 inhibitors was more potent in reducing the cell count of ML-1 cells than those of HL-60 (Figure 2C,D). Based on the data acquired from the analysis of leukemic cell viability and count, S63845 at a concentration of 0.1 µM for HL-60 cells and 5 μM for ML-1 cells was chosen for further analyses. In the case of ABT-737, a concentration of 5 μM was used for both AML cell lines.

### 2.2. Combination of S63845 and ABT-737 Induces Morphological Changes of AML Cells Indicating Cell Differentiation and Apoptosis

**Figure 3:**
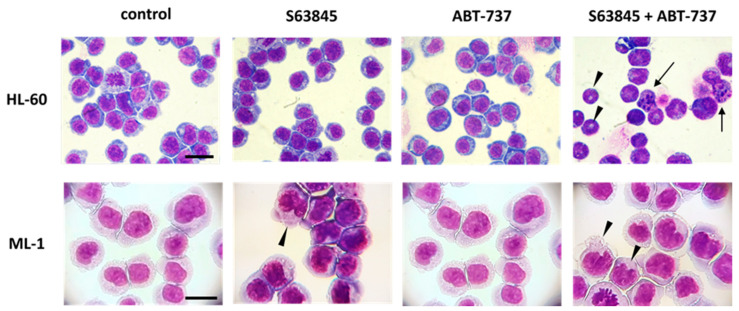
Morphological changes of AML cells following 48 h treatment with S63845 and ABT-737. Representative images of HL-60 and ML-1 cells stained with Hemacolor demonstrating cells with characteristic features of apoptosis (arrows) and differentiation (arrowheads). Scale bar = 20 µM.

To further evaluate the effects of S63845 on AML cells when used alone or in combination with ABT-737, we examined the morphological changes in the tested cell lines (Figure 3). The presence of apoptotic cells was confirmed by characteristic morphological features, such as cell shrinkage, membrane blebbing, condensation of chromatin and nuclear fragmentation [2]. These changes were rarely seen among HL-60 and ML-1 cell populations when they were treated with S63845 or ABT-737 separately but became distinctly visible after exposure to the combination of these agents (Figure 3). Moreover, the morphological features of differentiation, such as changes in cell size, alterations in the nuclear shape and in the cytoplasm staining, were observed among AML cells treated with the combination of S63845 and ABT-737. The exposure of HL-60 cells to both BCL-2 inhibitors added together resulted in changes characteristic of differentiation toward the granulocytic lineage, such as the appearance of condensed and lobulated nuclei, reduced cytoplasmic basophilia and reduced nuclear/cytoplasmic ratio. ML-1 cells, on the other hand, expressed morphology, which indicated their differentiation toward the monocytic lineage, including intended and irregular nuclei and less basophilic cytoplasm (Figure 3). Importantly, morphological features of differentiation were rarely seen in AML cells treated with S63845 or ABT-737 as a single agent.

### 2.3. Combination of S63845 and ABT-737 Enhances Differentiation and Apoptosis in AML Cells

**Figure 4:**
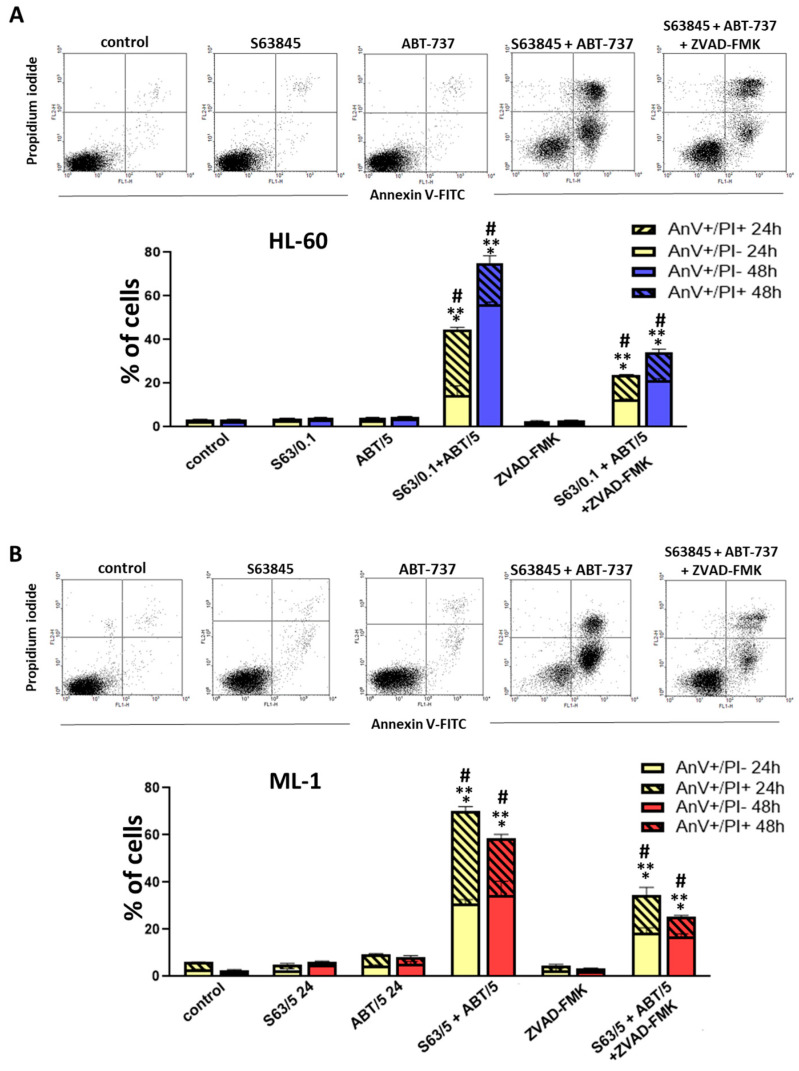
Effects of S63845 (S63) applied alone or in combination with ABT-737 (ABT) on apoptosis of AML cells. Apoptosis was determined in HL-60 (**A**) and ML-1 (**B**) cells by flow cytometry Annexin V-FITC/propidium iodide (PI) assay 24 h and 48 h after the agent application. (**A**,**B**) upper panels—the representative flow cytometry dot plots of Annexin V-FITC/PI staining obtained at the 48 h time point. Designations 0.1 and 5 indicate the agent concentrations (µM). Values are reported as the means ± SD of three independent experiments. Values significantly different (*p* ˂ 0.05) according to one-way ANOVA are designated by * and ** compared to, respectively, S63845 or ABT-737—single treated group; # compared to the corresponding control.

In order to confirm that S63845 combined with ABT-737 significantly increases apoptosis in HL-60 and ML-1 cells, we tested the ability of these BCL-2 inhibitors to trigger phosphatidylserine externalization and plasma membrane disruption. The combination treatment of AML cells for 24 h and 48 h significantly increased the percentage of annexin V+/PI− (early apoptotic) and annexin V+/PI+ (late apoptotic) cells compared with vehicle or single-agent treatments. At the 48 h time point, approximately 74% of HL-60 cells and 59% of ML-1 cells treated with a combination of BH3 mimetics were annexin V positive (all apoptotic cells) (Figure 4A,B). Further, we checked whether pretreatment of AML cells with ZVAD-FMK, a pan-caspase inhibitor, would reverse apoptosis induction in leukemic cells. It was found that ZVAD-FMK partly prevented HL-60 and ML-1 cell death induced by both agents’ combination (Figure 4A,B). These data demonstrated that the combination of S63845 and ABT-737 significantly induced apoptosis in AML cells.

**Figure 5:**
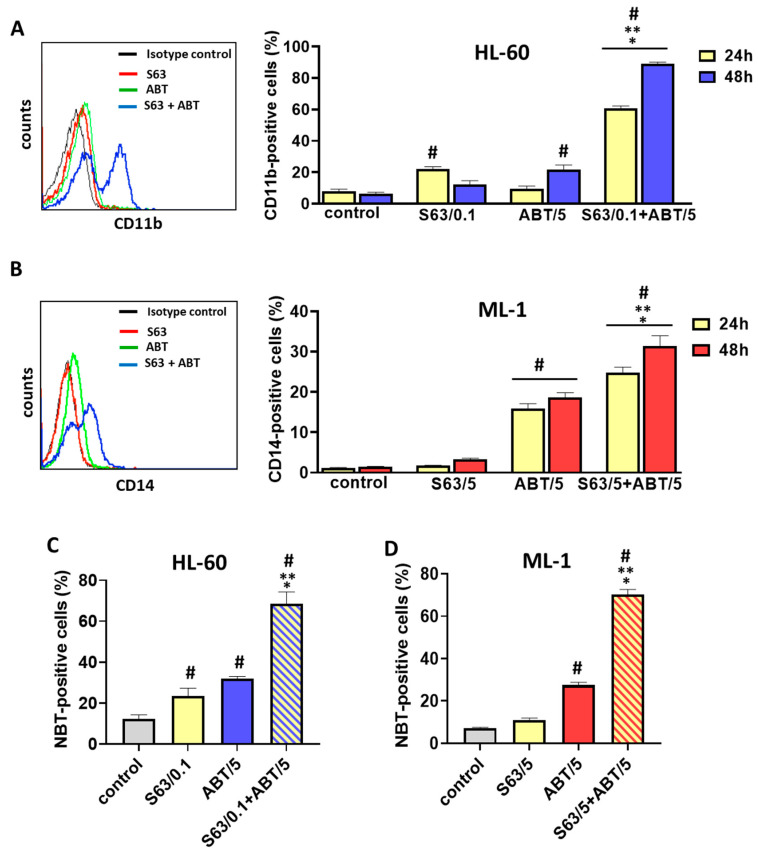
The effects of S63845 (S63) applied alone or in combination with ABT-737 (ABT) on differentiation of AML cells were evaluated 24 h and/or 48 h after cell exposure to the tested agents. (**A**) The percentages of CD11b-positive HL-60 cells and (**B**) the percentage of CD14-positive ML-1 cells were analyzed using flow cytometry. (**A**,**B**) left panels—the representative histograms of the expression of CD11b and CD14, respectively. Changes in the percentages of HL-60 (**C**) and ML-1 (**D**) cells performing oxidative burst (NBT-positive cells) were assessed at the 48 h time point using the NBT assay. Designations 0.1 and 5 indicate the agent concentrations (µM). Values are reported as the means ± SD of three independent experiments. Values significantly different (*p* ˂ 0.05) according to one-way ANOVA are designated by * and ** compared to, respectively, S63845 or ABT-737—single treated group; # compared to the corresponding control.

To further verify the pro-differentiating effect of S63845 in combination with ABT-737 on HL-60 and ML-1 cells, we analyzed the cell surface expression of CD11b and CD14, respectively, and determined the ability of these cells to produce superoxide and reduce NBT. CD11b is the αM protein subunit for the β2-integrin and is considered to be a specific marker of HL-60 cell differentiation to the granulocytic lineage [28]. CD14 antigen was originally described as a differentiation antigen on mononuclear cells and is a common marker of the monocyte lineage [29]. As indicated in Figure 5A, the exposure of HL-60 cells to S63845 in combination with ABT-737 significantly increased the number of cells expressing the CD11b antigen compared to the control and single-agent treatments. Similarly, the percentage of ML-1 cells expressing CD14 significantly increased when S63845 and ABT-737 were applied together (Figure 5B). Next, we determined the percentage of NBT-positive HL-60 and ML-1 cells as a result of such treatment. The distinctly higher number of NBT-positive cells was observed when AML cells were exposed to S63845 in combination with ABT-737 (Figure 5C,D).

### 2.4. S63845 Alters the Expression of MCL-1 on Protein Level When Given in Combination with ABT-737

**Figure 6:**
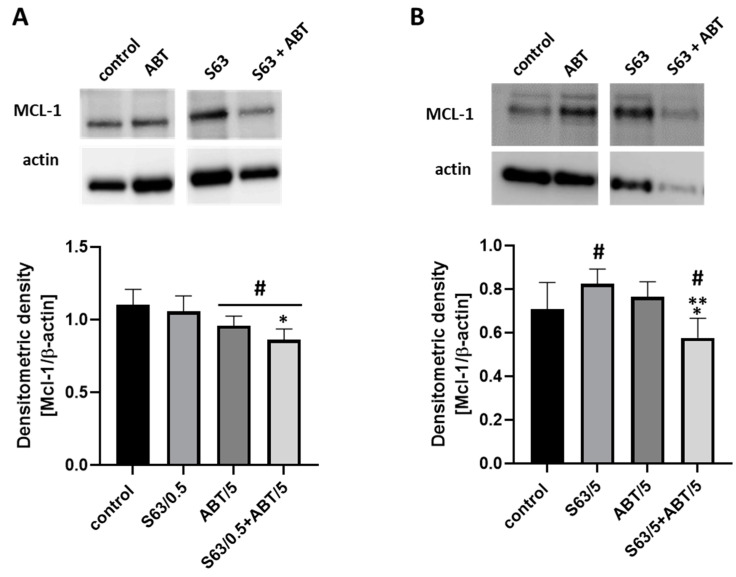
Expression of MCL-1 protein in HL-60 (panel **A**) and ML-1 (panel **B**) cells after treatment with S63845 (S63) and ABT-737 (ABT) alone and in combination for 48 h, detected by Western blotting. Relative levels of MCL-1 protein were quantified by densitometry and normalized to β-actin. Designations 0.5 and 5 indicate the agent concentrations (µM). Values are reported as the means ± SD of three independent experiments. Values significantly different (*p* ˂ 0.05) according to one-way ANOVA are designated by * and ** compared to, respectively, S63845 or ABT-737—single treated group; # compared to the corresponding control.

Given the key role of the MCL-1 protein in hematopoietic cell differentiation and apoptosis, we verified MCL-1 expression in HL-60 and ML-1 cells exposed for 48 h to S63845 as a single agent and in combination with ABT-737. Compared to the control group, HL-60 cells exposed to S63845 alone exhibited a similar level of MCL-1 expression (Figure 6A), whereas in ML-1 cells, its level increased (Figure 6B). The results of Western blot analyses revealed that the combination of S63845 and ABT-737 decreased the expression of the MCL-1 protein in HL60 and ML-1 cells as compared to the single-inhibitor-treated group (Figure 6A,B).

### 2.5. MAPK Pathway Inhibition Potentiates the Pro-Apoptotic and Pro-Differentiating Effects of S63845 in AML Cells

**Figure 7:**
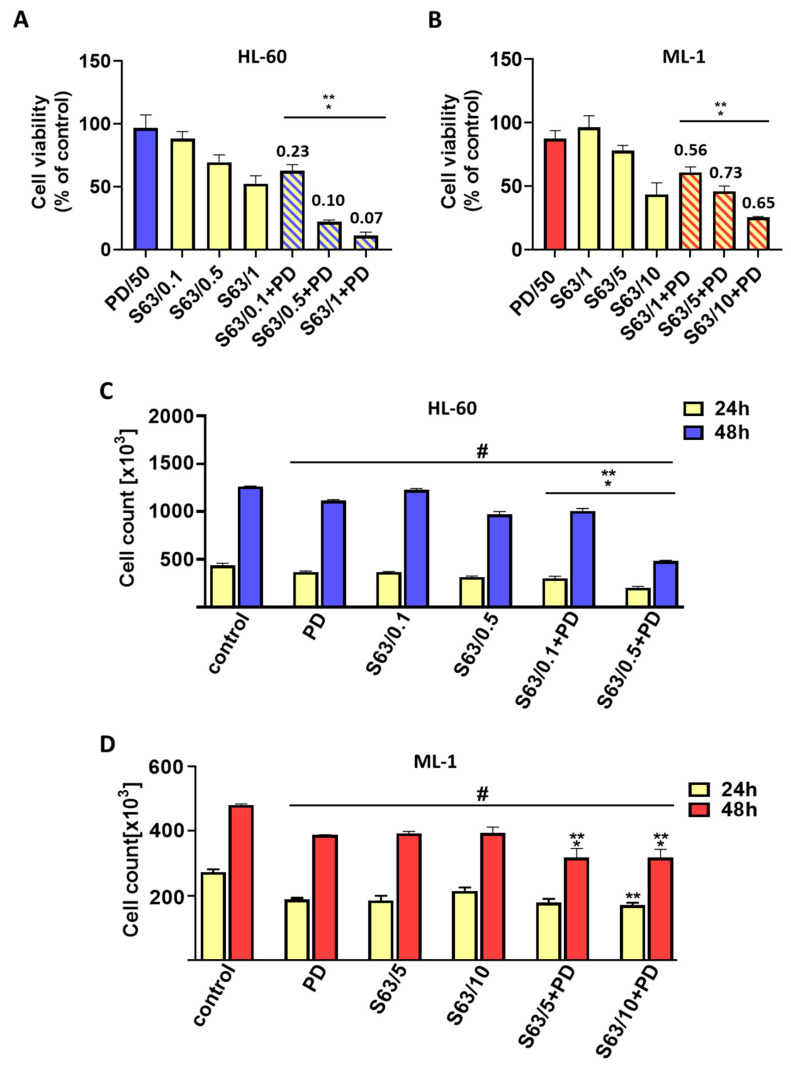
Effects of S63845 (S63) and MAPK inhibitor PD98059 (PD) given as single agent or in combination on the viability and count of AML cells. Using PrestoBlue assay, the cell viability of HL-60 (**A**) and ML-1 (**B**) cells was determined at 48 h. The synergism between the tested agents was determined by the combination index (CI) analysis at a non-constant ratio. The CI values, generated using the CompuSyn software according to the Chou–Talalay method, are given in graph (**A**,**B**). Using the Coulter method, the HL-60 (**C**) and ML-1 (**D**) cell count analyses were performed at 24 h and 48 h after application of the tested agents. Designations 0.1, 0.5, 5 and 10 indicate the S63845 concentrations (µM). Three independent experiments were performed in triplicate (**A**,**B**) or duplicate (**C**,**D**). Values are reported as the means ± SD. Values significantly different (*p* ˂ 0.05) according to one-way ANOVA are designated by * and ** compared to, respectively, S63845 or PD98059—single treated group; # compared to the corresponding control.

Considering the importance of MAPK signaling pathway in the regulation of MCL-1 function as well as leukemic cell growth and survival, we studied the effects of MAPK cascade inhibition using its highly selective in vitro inhibitor PD98059. The inhibition of MAPK phosphorylation by PD98059 was confirmed by Western blot analysis. A decrease in the level of phosphorylated MAPK in relation to total MAPK level was observed in HL-60 cells after 1 h and 2 h exposure to PD98059 and in ML-1 cells after a 2 h treatment (Supplementary Figure S2). Cell viability assays showed that MAPK inhibition in AML cells significantly sensitized cells to the MCL-1 inhibitor. The combination of PD98059 and S63845 was very potent in reducing the cell viability of both AML cell lines; however, a more pronounced effect was seen in HL-60 cells (Figure 7A,B). The synergism of the agent combination was confirmed by the isobologram and combination index analysis. The CI values in HL-60 cells ranged from 0.07 to 0.23, and from 0.56 to 0.65 in ML-1 cells, indicating strong synergism, especially in HL-60 cells (Figure 7A,B, Supplementary Figure S3). These observations were further verified by the measurement of the cell count. Coulter counter analysis revealed the significant decrease in the number of HL-60 and ML-1 cells pretreated with a MAPK inhibitor and exposed to S63845 compared with the values obtained in the control group and after a single application of these compounds. The reduction in HL-60 count seemed to be more prominent than that observed in ML-1 cells (Figure 7C,D).

**Figure 8:**
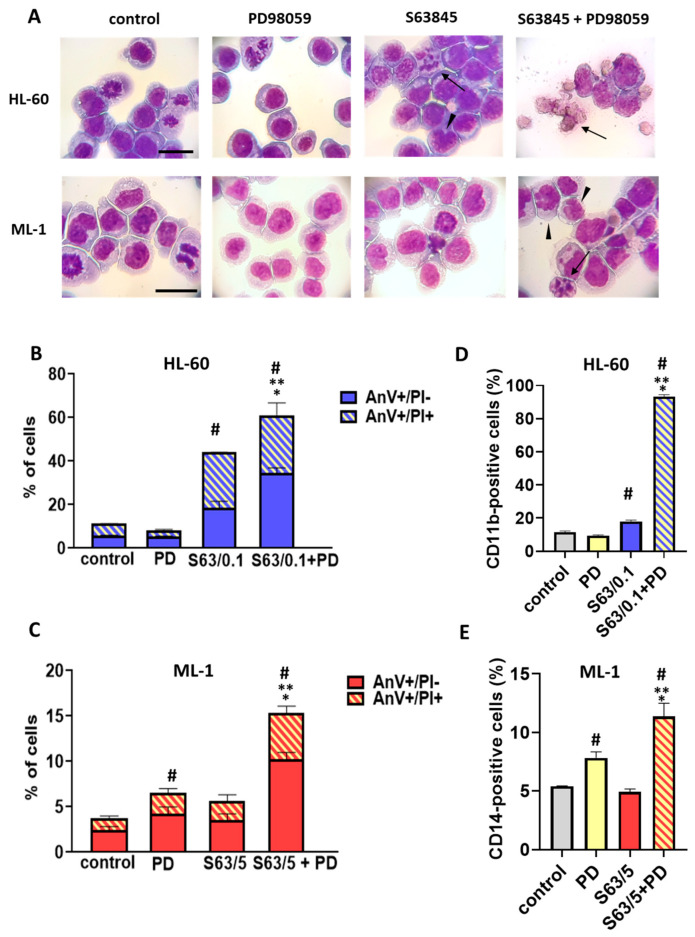
Effects of S63845 (S63) and MAPK inhibitor PD98059 (PD) applied alone or in combination on apoptosis and differentiation of AML cells. (**A**) Representative micrographs of leukemic cells stained with Hemacolor demonstrating cells with characteristic features of apoptosis (arrows) and differentiation (arrowheads) (original magnification 1000×, scale bar = 20 µm). Apoptosis was determined in HL-60 (**B**) and ML-1 (**C**) cells by flow cytometry Annexin V-FITC/propidium iodide assay 48 h after agent application. The effects of S63845 and PD98059 on the differentiation of AML cells were evaluated after 48 h cell exposure to the tested agents. (**D**) The percentages of CD11b-positive HL-60 cells and (**E**) the percentage of CD14-positive ML-1 cells were analyzed using flow cytometry. Designations 0.1 and 5 indicate the S63845 concentrations (µM). Values are reported as the means ± SD of three independent experiments. Values significantly different (*p* ˂ 0.05) according to one-way ANOVA are designated by * and ** compared to, respectively, S63845 or PD98059—single treated group; # compared to the corresponding control.

We then determined whether the inhibition of MAPK signaling enhances the pro-apoptotic activity of S63845 in AML cells. The microscopic analysis of AML cells clearly showed an increase in AML cells with apoptotic features after combined inhibition of MAPK and MCL-1. Importantly, a distinctly higher frequency of apoptotic cells and apoptotic bodies was observed among HL-60 cells than among ML-1 cells (Figure 8A). This observation was confirmed by flow cytometric analysis using annexin V-FITC/propidium iodide assay. The inhibition of MAPK alone did not increase apoptosis in AML cells. However, PD98059 significantly potentiated the effects of S63845 relative to the levels of apoptosis observed in vehicle-treated and single-agent-treated HL-60 and ML-1 cells. Moreover, HL-60 cells appeared to be more susceptible to simultaneous inhibition of the MAPK cascade and MCL-1 protein than ML-1 cells, as the percentage of annexin V positive cells (all apoptotic cells) was approximately 60% and 15% among HL-60 and ML-1 cell populations, respectively (Figure 8B,C).

The influence of MAPK pathway inhibition on the pro-differentiating activity of S63845 was further explored in AML cells through analysis of the surface expression of differentiation markers. Pre-treatment of HL-60 cells with PD98059 significantly increased the percentage of CD11b-positive cells after exposure to S63845 (Figure 8D), whereas the number of CD14-positive ML-1 cells was only slightly, although significantly, enhanced (Figure 8E).

### 2.6. Combination of MAPK Inhibitor and S63845 Alters the Expression of MCL-1 Protein

**Figure 9:**
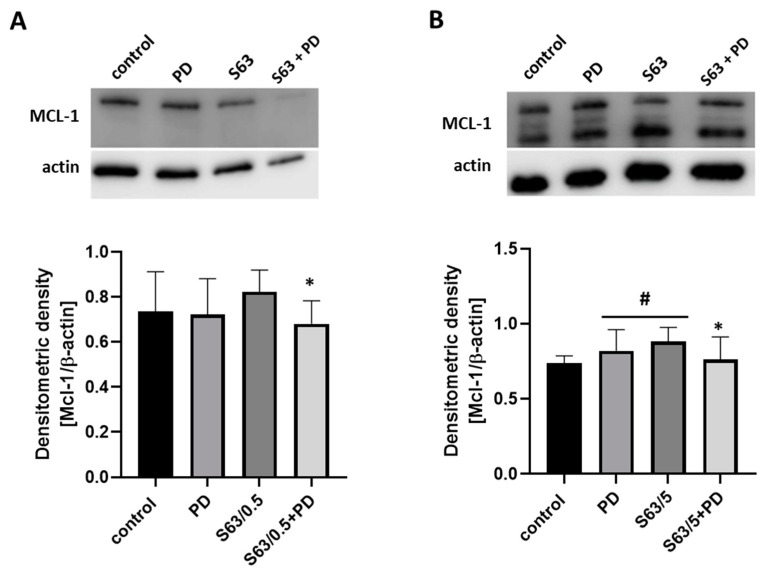
Expression of MCL-1 protein in HL-60 (panel **A**) and ML-1 (panel **B**) cells after treatment with S63845 (S63) and PD98059 (PD) alone and in combination for 24 h, as detected by Western blotting. Relative levels of MCL-1 protein were quantified by densitometry and normalized to β-actin. Designations 0.5 and 5 indicate the S63845 concentrations (µM). The results are expressed as the mean values ± SD of three independent experiments. Values significantly different (*p* ˂ 0.05) according to one-way ANOVA are designated by * compared to S63845—single treated group; # compared to the corresponding control.

To identify whether the observed changes induced by S63845 and PD98059 in AML cells were accompanied by alteration in MCL-1 protein level, Western blot analyses were performed. As shown in Figure 9A, the exposure of HL-60 cells to a MAPK inhibitor had no impact on the expression of MCL-1 protein, whereas a combination of S63845 with PD98059 resulted in significant reduction in MCL-1 protein level in comparison to its expression found in the S63845 single-agent-treated groups. Interestingly, in ML-1 cells, S63845 alone induced a slight but significant increase in MCL-1 protein level, which was diminished when leukemic cells were pretreated with PD98059 (Figure 9B).

## 3. Discussion

Targeted therapy has gained much interest in cancer treatment since the introduction of the first targeted drugs in oncology, such as all-trans retinoic acid (ATRA) for acute promyelocytic leukemia (APL) [30] and BCR-ABL1 tyrosine kinase inhibitor, imatinib, which has been the mainstay of chronic myeloid leukemia (CML) therapy for decades [31]. In the past 20 years, there has been a significant increase in FDA-approved targeted drugs for cancer treatment. Nevertheless, their development is still challenging. In this context, the discovery of therapeutic targets is of key importance. The recognition of the pivotal role played by anti-apoptotic BCL-2 family proteins in cancer development and treatment resistance made them a relevant target for anticancer therapy [32]. Indeed, venetoclax, the first BCL-2 inhibitor approved by the FDA, is a mild and efficient drug for treating chronic lymphocytic leukemia and acute myeloid leukemia, especially when combined with other anticancer agents [33]. However, the responses to venetoclax in various leukemia subtypes can differ significantly. Failures of BCL-2 inhibition may rely on the survival dependence of cancer cells on numerous other anti-apoptotic proteins. Brunelle et al. [34] compared the function of MCL-1 and BCL-2 in cancer cell survival using mouse leukemia models based on Eμ-Myc expression, where either BCL-2 or MCL-1 were required for leukemia maintenance. It was shown that leukemias are dependent on the continuous function of either MCL-1 or BCL-2 for survival. Accumulating evidence has indicated the critical pro-survival role of MCL-1 in hematological malignancies, making this protein an important target in leukemia therapy [20]. In line with this, herein, we utilized two myeloid leukemia cell lines to study the antileukemic efficacy of a novel MCL-1 inhibitor, S63845. This study showed that it exerts potent antileukemic activity and synergizes with BCL-2/BCL-XL inhibitor ABT-737 to induce apoptosis and differentiation in AML cells. Moreover, it was found that the pro-apoptotic and pro-differentiating activity of S63845 in AML cells might be enhanced by the inhibition of MAPK signaling pathway.

The results of the present as well as previous studies demonstrated that S63845, used as a single agent, decreased AML cell viability and count in a concentration- and cell-line-dependent manner [23,35]. In terms of cell viability, it was shown that among a panel of cancer cell lines derived from hematologic malignancies and solid tumors, the MCL-1 inhibitor appeared to be the most potent in AML and multiple myeloma, showing IC50 values of <1 µM [23]. However, the IC50 value of S63845 for both AML cell lines tested in the present study was above 1 µM, indicating their resistance to the MCL-1 inhibitor. As shown by Western blot analysis, both HL-60 and ML-1 cells expressed MCL-1 at a comparable level, and S63845 was able to enhance the level of this protein. Interestingly, in ML-1 cells, two isoforms of MCL-1 were noticed at the 24 h time point. As shown by numerous studies in humans, Mcl-1 mRNA may undergo alternative splicing to produce two isoforms of protein with opposite functions. The long variant, MCL-1L (40 kDa), acts as an anti-apoptotic factor and is traditionally referred to as MCL-1, whereas the short variant, MCL-1S (32 kDa), displays pro-apoptotic features [24]. Splicing dysregulation of MCL-1 may lead to resistance toward apoptosis and is observed in various types of cancers [24]. Given that two isoforms were seen in ML-1 cells but not in HL-60 cells, this may indicate the important role of mRNA splicing in ML-1 cell survival and response to treatment. Further detailed mechanistic studies are required to resolve this issue. Importantly, inhibition of the MCL-1 protein by a specific inhibitor did not always result in a reduction in its level. No changes in the expression of MCL-1 were found in the sensitive AML cell line MV4-11 treated with S63845 [36]. Interestingly, MCL-1 inhibitor can cause an increase in total MCL-1 protein level, which was observed in difficult-to-treat melanoma cells [37], and it was also found in the present study in ML-1 cells treated with S63845. In previous studies, this was hypothesized to be due to the compound displacement of NOXA, a BH3 protein involved in MCL-1 turnover. Indeed, it was shown by the numerous studies that the mechanism of S63845 action in cancer cells is correlated with the release of pro-apoptotic proteins, such as NOXA, BAK and BIM, upon S63845 binding to the MCL-1 protein [38]. On the other hand, sensitivity to S63845 was correlated with diminished expression of other anti-apoptotic proteins in cutaneous T-cell lymphoma cells [39], melanoma [37] and myeloma cells [40]. In particular, MCL-1 inhibition may lead to BCL-XL overexpression in cancer cells as a compensatory survival adaptation. In line with this, in the present study, we explored the effects of combined treatment covering both MCL-1 and BCL2/BCL-XL inhibition.

Our data indicate that treatment of AML cells with a combination of S63845 and ABT-737, a well-defined BCL-2/BCL-XL inhibitor, resulted in more effective cell killing compared to single-agent treatment. The analysis of the combination index clearly showed a strong synergism of the S63845 and ABT-737 interaction. We found a pronounced reduction in cell viability and count as well as an increase in the percentage of apoptotic cells, as shown by the annexin V/propidium iodide assay and morphological examination. Importantly, the use of ZVAD-FMK, a pan-caspase inhibitor, markedly diminished apoptosis triggered by the combined treatment, indicating that the antileukemic effect of S63845 and ABT-737 applied in combination might primarily stem from the activation of the apoptotic pathway. In addition, S63845 together with ABT-737 was able to reduce the MCL-1 protein level. The high pro-apoptotic efficacy of MCL-1 and BCL-2/BCL-XL co-inhibition was also found in previous studies concerning hematological malignancies and solid tumors [37,41]. Decreased viability of cutaneous T-cell lymphomas in response to ABT-737 was shown to be further reduced in combinations with S63845 [39]. The use of S63845 with ABT-263, an orally available analog of ABT-737, induced apoptosis in melanoma cells with high potency, both in vitro and in vivo [37]. Of note, the combination of S63845 with either selective BCL-2 or BCL-XL inhibitor also resulted in synergistic reduction in cervical cancer cell growth and invasion [41], increased apoptotic response in AML cell lines and primary samples [36], and exerted profound synergistic activity against T-cell acute lymphoblastic leukemia cells (T-ALL) in vitro and in vivo in a T-ALL zebrafish embryos model [42]. Taken together, the results of the present study as well as previous investigations clearly show the high potency of combined inhibition of anti-apoptotic BCL-2 family members in cancer cells. Of note, co-inhibition with BCL-2 inhibitors requires lower concentrations of them compared to the doses needed to assess their pro-apoptotic activity in the single-agent treatments. In this way, the therapeutic index of BCL-2 inhibitors can be enhanced with concomitant decrease in potential adverse effects. For example, ABT-737 and ABT-263 by themselves cause dose-dependent thrombocytopenia as a result of BCL-XL inhibition. By lowering the dosage of BCL-XL inhibitors, this side effect of their use is expected to be markedly decreased.

In addition to the above observations of pronounced pro-apoptotic activity of S63845 and ABT-737 combinational treatment of AML cells, we report for the first time the pro-differentiating impact of this treatment. HL-60 and ML-1 cells exposed to S63845 in combination with ABT-737 acquired a phenotype characteristic of more mature myeloid cells, such as unique morphology, the ability to perform oxidative burst and higher expression of a specific differentiation marker. In particular, HL-60 cells displayed a morphology of granulocytic lineage cells with enhanced expression of CD11b, whereas the expression of CD14 and morphology of ML-1 cells indicated their monocytic differentiation. Previous studies have demonstrated that the BCL-2 protein family is crucial for both controlled myelopoiesis and homeostasis of mature myeloid cells. Interestingly, during differentiation, AML cells undergo a number of marked changes in apoptosis-related genes, among which a down-regulation of the BCL-2 protein appears to be a part of the differentiation pathway and may serve to facilitate the apoptotic response [43]. It was shown that differentiation and consequent apoptosis of HL-60 cells in response to ATRA treatment was accompanied by bcl-2-mRNA instability [44]. In contrast, MCL-1 is crucial for the survival of myeloid precursor cells and their successful maturation into granulocytes [45]. A conditional knockout of MCL-1 in the myeloid lineage led to disturbed neutrophil differentiation with an increase in immature precursors’ number and a general deficit of mature granulocytes [45]. On the other hand, differentiation into monocytes is not disturbed by the down-regulation of MCL-1, showing increased expression of BCL-2 and BCL-XL [45]. In the present study, among the two cell lines tested, ML-1 cells in particular differentiated in response to ABT-737 alone, whereas both AML cell lines showed clear features of differentiation after combined treatment with S63845 and ABT-737. Based on this observation, it can be assumed that induction of AML cell differentiation and consequent apoptosis may be achieved by simultaneous inhibition of both MCL-1 and BCL-2 proteins. In the previous study, regarding the pro-differentiating mechanisms of ATRA in NB4 and HL-60 cells, it was shown that ATRA increased the level of MCL-1 protein, while BCL-2 expression was decreased. Inhibition of MCL-1 appeared to accelerate the apoptosis of the ATRA-differentiated AML cells [46]. In line with this, additional experiments are needed to firmly establish the role of anti-apoptotic BCL-2 family proteins in differentiation of AML cells induced by combined treatment with S63845 and ABT-737. Nevertheless, it is clear now that the close relationships between the processes mediating apoptosis and differentiation are fundamental to leukemia and are thus important for the development of an effective targeted therapy.

When considering the enhancement of S63845 antileukemic activity, the MAPK signaling pathway is also of importance. This pathway is associated with an apoptosis-resistant phenotype due to the anti-apoptotic function of MAPK signaling. It was found that in primary AML cells, MAPK is constitutively active and promotes leukemic cell growth [5]. With regard to the MCL-1 protein, the activation of the MAPK pathway leads to its increased stability and higher levels [47]. Simultaneous up-regulation of the BCL2 ratio and activation of the MAPK pathway act synergistically to enhance leukemic cell survival. Therefore, in the present study, in relation to the antileukemic effects of S63845, we also report that inhibition of the MAPK pathway can enhance the pro-apoptotic as well as pro-differentiating activity of this MCL-1 inhibitor in AML cells. We found a strong synergism of the S63845 and PD98059 combination, which was confirmed by combination index analysis. A synergistic effect of combined ERK1/2 and MCL-1 inhibition was also seen in rhabdomyosarcoma cell lines [48]. Moreover, the sensitivity of AML cells to co-treatment with S63845 and MEK inhibitor trametinib was greater in leukemic cells with elevated MCL-1 and MEK levels [47]. Interestingly, previous mechanistic studies revealed that the ERK1/2 signaling pathway is activated in response to venetoclax treatment, which contributes to an acquired resistance phenotype [49]. The observed response of cancer cells to the combinational treatment with BCL-2 and MAPK inhibitors may be related to the phenomenon of cancer cell dependence on a specific oncogene to sustain growth and survival. In this way, the inhibition of this specific oncogene/pathway seemed to be sufficient to inhibit the neoplastic phenotype. Importantly, as shown in the present study, the inhibition of MAPK phosphorylation may occur in the first hours after cell exposure to the MAPK inhibitor.

In conclusion, our study reveals that targeting different anti-apoptotic proteins from the BCL-2 family simultaneously by specific inhibitors leads to profound reduction in AML cell growth and, equally important, their differentiation. The observed pro-apoptotic and pro-differentiating activity of S63845 enhanced by the combination with BCL-2/BAX or MAPK inhibitors provides the rationale for further studies regarding the use of this strategy in AML therapy.

## 4. Materials and Methods

### 4.1. Reagents

S63845, ABT-737 and ZVAD-FMK were purchased from Selleck Chemicals (Munich, Germany). BCL-2 inhibitors were dissolved in DMSO and stored as 5 mM stock solutions at −20 °C. RPMI 1640 medium, fetal calf serum and phosphate-buffered saline (PBS) were obtained from GIBCO BRL Life Technologies (Gaithersburg, MD, USA). Antibiotic antimycotic solution (AAS), dimethyl sulfoxide (DMSO), Nitroblue tetrazolium, phorbol 12-myristate 13-acetate, PD98059 were purchased from Sigma Aldrich (St. Louis, MO, USA). PrestoBlue™ Cell Viability reagent was acquired from Invitrogen (Life Technologies, Eugene, OR, USA). FITC Annexin V Apoptosis Detection Kit I, propidium iodide (PI)/RNase staining buffer, anti CD11b and anti-CD-14 were obtained from BD Biosciences (BD Biosciences, San Diego, CA, USA). CellEvent™ Caspase 3/7 Green Flow Cytometry Assay kit was purchased from Molecular Probes (Eugene, OR, USA). The Hemacolor^®^ Rapid Staining kit was obtained from Merck Millipore (Darmstadt, Germany). RIPA lysis buffer and Halt™ Protease Inhibitor Cocktail were obtained from ThermoScientific (Rockford, IL, USA). The antibody against MCL-1 (cat. #4572), pMAPK (cat. #9101), MAPK (cat. #9102), β-actin (cat. #4970) and horseradish peroxidase-conjugated secondary antibody (cat. #7074) were purchased from Cell Signaling Technology (Danvers, MA, USA). WesternBright Sirius Western blotting HRP substrate was acquired from Advansta (Menlo Park, CA, USA).

### 4.2. AML Cell Culture and Treatment

Human acute myeloid leukemia cell lines HL-60 (ATCC, Manassas, VA, USA) and ML-1 (ECACC, Salisbury, UK) were maintained in RPMI 1640 medium supplemented with 10% fetal calf serum, 2 mM L-glutamine and AAS containing 20 units of penicillin, 20 mg streptomycin and 0.05 mg amphotericin B. The cells were passaged every 2–3 days. The cells grew exponentially at 37 °C in an atmosphere of 5% CO_2_ in air (HERAcell incubator, Thermo Fisher Scientific, Waltham, MA, USA). Leukemic cells were seeded in 12-, 24- or 96-well plates at a density of 1 × 10^5^ cells/mL prior to performing the experiments. For cell viability analysis, HL-60 and ML-1 cells were treated with S63845 at a concentration range of 0.01–10 µM. For subsequent experiments, adequate S63845 concentrations were chosen for each cell line based on the results of the cell viability assay. The concentrations of ABT-737 were chosen based on previous studies [27]. The BH3 mimetic concentrations were physiologically relevant and could potentially be achieved in vivo. Leukemia cells were treated with a single inhibitor or simultaneous combination of S63845 and ABT-737 for 24 h or 48 h. The control treatments consisted of untreated and DMSO-treated HL-60 and ML-1 cells. The final concentration of DMSO did not influence the analyzed parameters, and no significant differences in cell response to this diluent were observed; therefore, the data obtained from DMSO-treated cells were considered as control. To study the effects of S63845 in combination with MAPK inhibitor, the cells were pretreated with 50 µM of PD98059 for 1 h, then treated with S63845 for an additional 24 h or 48 h. In some experiments, AML cells were pre-incubated with a pan-caspase inhibitor Z-VAD-FMK for 1 h.

### 4.3. Analysis of AML Cell Viability and Count

Cell viability was assessed using the PrestoBlue reagent (PB) according to the manufacturer’s protocol. After 48 h exposure of HL-60 and ML-1 cells to BCL-2 and/or MAPK inhibitors, 11 μL of PB solution was added to the wells containing 100 μL of cell suspension, and the plate was incubated in darkness for 60 min at 37 °C. The fluorescence of resorufin was measured using TECAN Infinite F200 PRO plate reader with excitation and emission wavelengths set at 535 nm and 595 nm, respectively. Cell viability was expressed as a percentage relative to control. The IC50 (half-maximal inhibitory concentration) values for S63845 were calculated from the concentration–response curves.

The number of HL-60 and ML-1 cells was analyzed using a Z2 Coulter counter (Beckman Coulter, INC, Fullerton, CA, USA), as previously described in detail [50]. The cell counts were determined at a range of 476–2432 fL using Z2 AccuComp software version 3.0 (Beckman Coulter INC, USA).

### 4.4. Morphological Observation of AML Cells

To assess the morphology changes in HL-60 and ML-1 cells, staining using Hemacolor was performed. Briefly, approximately 1 × 10^5^ cells were resuspended in 0.2 mL PBS. The cell suspension was added to the cytospin chamber and centrifuged at 1000 rpm for 6 min at 4 °C. After air drying, the prepared cytospins were fixed in methanol for 15 min at room temperature. Then, each slide was stained with the Hemacolor^®^ Rapid Staining Kit according to the manufacturer’s instructions. The cellular morphology was analyzed using a light microscope under 100× magnification (AXIO Scope.A1 microscope, Carl Zeiss Microscopy, Oberkochen, Germany).

### 4.5. Annexin V-FITC/PI Assay

To determine the induction of apoptosis, dual staining of HL-60 and ML-1 cells with fluoresceinated annexin V (annexin V-FITC) and propidium iodide (PI) was performed using FITC Annexin V Apoptosis Detection Kit I. After 24 h and 48 h of cell incubation with the tested agents, the cells were harvested and then washed twice with cold PBS. The cell pellets were resuspended in 100 μL of binding buffer and 5 µL of annexin V-FITC, and 5 μL of PI staining solution was added to the cell suspension. Cells were incubated in the dark for 15 min at room temperature. Following the incubation, 400 μL of the binding buffer was added to each tube, and cells were analyzed using the FACS Calibur flow cytometer (Becton Dickinson). Two-parameter fluorescence dot plots were generated by analyzing at least 1 × 10^4^ cells. The percentages of annexin V positive/PI negative cells and annexin V positive/PI positive cells were determined using WinMDI software version 2.8.

### 4.6. Nitroblue Tetrazolium Assay

Nitroblue tetrazolium (NBT) was used to estimate intracellular O_2^−^_ as a measure of differentiation of HL-60 and ML-1 cells. For this assay, AML cells were seeded in a 96-well plate in a 100 µL final volume of cell suspension. At the indicated time point, phorbol 12-myristate 13-acetate (PMA) at a final concentration of 100 nM was added to each well to stimulate the cells. After cell incubation with PMA at 37 °C for 60 min, 10 μL of NBT solution (10 mg/mL) was added to each well, and the cells were further incubated at 37 °C for 60 min. Following the incubation period, the cytospin slides were prepared. A light microscope was used to identify NBT-positive cells, which contained intracellular blue formazan deposits. A total of 200 cells on each slide were counted, and the percentage of NBT-positive cells was calculated for each group.

### 4.7. Flow Cytometric Detection of CD11b and CD14 Expression

Quantitative immunofluorescence measurements were performed to determine the expression of myeloid differentiation markers CD11b and CD14 on HL-60 and ML-1 cells, respectively. AML cells were centrifuged, washed and resuspended in 100 μL of PBS. Then, 5 μL of either APC-conjugated CD11b antibody or FITC-conjugated anti-human CD14 antibody was added to the suspension, followed by incubation in the dark for 30 min at room temperature. After the incubation period, 400 μL of PBS was added to each tube, and the cells were centrifuged. Finally, the cells were resuspended in 500 μL of PBS and measured in the FACSCalibur flow cytometer. One-parameter fluorescence histograms were generated by analyzing at least 1 × 10^4^ cells in each sample. The percentages of CD11b-positive cells and CD14-positive cells were determined using BD CellQuest Pro software.

### 4.8. Preparation of Cell Lysates and Western Blot Analysis

Whole cell lysates were prepared using the RIPA lysis buffer supplemented with protease inhibitors. HL-60 and ML-1 cells were washed with ice-cold PBS, lysed on ice for 15 min and cleared by centrifugation at 15,000× *g* at 4 °C for 15 min. The protein content was determined via Bradford protein assay using bovine serum albumin (BSA) as a standard. Proteins (30 μg from each experimental group) were separated by Mini-PROTEAN^®^ TGX™ gels (Cat. No. 456–1093; BioRad, Hercules, CA, USA) using SDS-PAGE (BioRad Mini-PROTEAN II Electrophoresis Cell), followed by transferring them to PVDF membranes. The membranes were blocked in 5% BSA/TBST and probed with anti-MCL-1 primary antibody (1:1000 in blocking buffer) at 4 °C overnight. Next, the membranes were incubated with a horseradish peroxidase-conjugated secondary antibody diluted to 1:1000. The bands were detected by chemiluminescence using the WesternBright Sirius Western blotting HRP substrate (Advansta, Menlo Park, CA, USA), visualized using a ChemiDoc-It Imaging System (UVP, LLC, Cambridge, UK) and quantified using ImageJ analysis software (US National Institute of Health, Bethesda, MD, USA). The blots were then stripped and probed for anti-β-actin used as an internal control for the samples.

### 4.9. Statistical Analysis and Calculation of Synergy

The obtained results were confirmed by three independent experiments, and in the case of cell viability and count analysis, each experiment was performed in triplicate and duplicate, respectively. All data are shown as the mean value ±the standard deviation (SD). STATISTICA 10 software (StatSoft, Kraków, Poland) was used to perform statistical analyses. The statistical significance of the data was evaluated by one-way analysis of variance (ANOVA), followed by Tukey’s honestly significant difference multiple range test. A *p*-value < 0.05 was considered statistically significant. The agent interactions were analyzed using CompuSyn software version 1.0 (ComboSyn Inc., Paramus, NJ, USA). The combination index (CI) values were calculated according to the Chou and Talalay mathematical model for drug interactions [51]. CI < 1.0 represents synergy, CI = 1.0 indicates an additive effect, whereas CI > 1.0 implies antagonism.

## Data Availability

All data are contained within the manuscript.

## Supplementary Materials

The following supporting information can be downloaded at: https://www.mdpi.com/article/10.3390/ijms24087180/s1.
