# Supplementary material for: Synergistic Action of MCL-1 Inhibitor with BCL-2/BCL-XL or MAPK Pathway Inhibitors Enhances Acute Myeloid Leukemia Cell Apoptosis and Differentiation
Source: Int J Mol Sci. 2023 Apr 13;24(8):7180. doi: 10.3390/ijms24087180 (PMC10138770; doi:10.3390/ijms24087180)
Supplement: Supplementary file 1 [file ijms-24-07180-s001.zip › ijms-2222930-supplementary.pdf]

*Supplementary Material*

# Synergistic Action of MCL-1 Inhibitor with BCL-2/BCL-XL or MAPK Pathway Inhibitors Enhances Acute Myeloid Leukemia Cell Apoptosis and Differentiation

**Małgorzata Opydo [1,*], Anna Mlyczyńska [1], Ewa Mlyczyńska [2,3], Agnieszka Rak [2] and Elzbieta Kolaczkowska [1,*]**

[1] Laboratory of Experimental Hematology, Institute of Zoology and Biomedical Research, Faculty of Biology, Jagiellonian University, 30-387 Krakow, Poland
[2] Laboratory of Physiology and Toxicology of Reproduction, Institute of Zoology and Biomedical Research, Faculty of Biology, Jagiellonian University, 30-387 Krakow, Poland
[3] Doctoral School of Exact and Natural Sciences, Jagiellonian University, 30-387 Krakow, Poland
* Correspondence: malgorzata.opydo@uj.edu.pl (M.O.); ela.kolaczkowska@uj.edu.pl (E.K.); Tel.: +48-12-664-52-29 (M.O.); +48-12-664-50-65 (E.K.)

*2.1. S63845 exerts cytotoxic effect on AML cells and synergizes with ABT-737 in reducing leukemic cell viability and count*

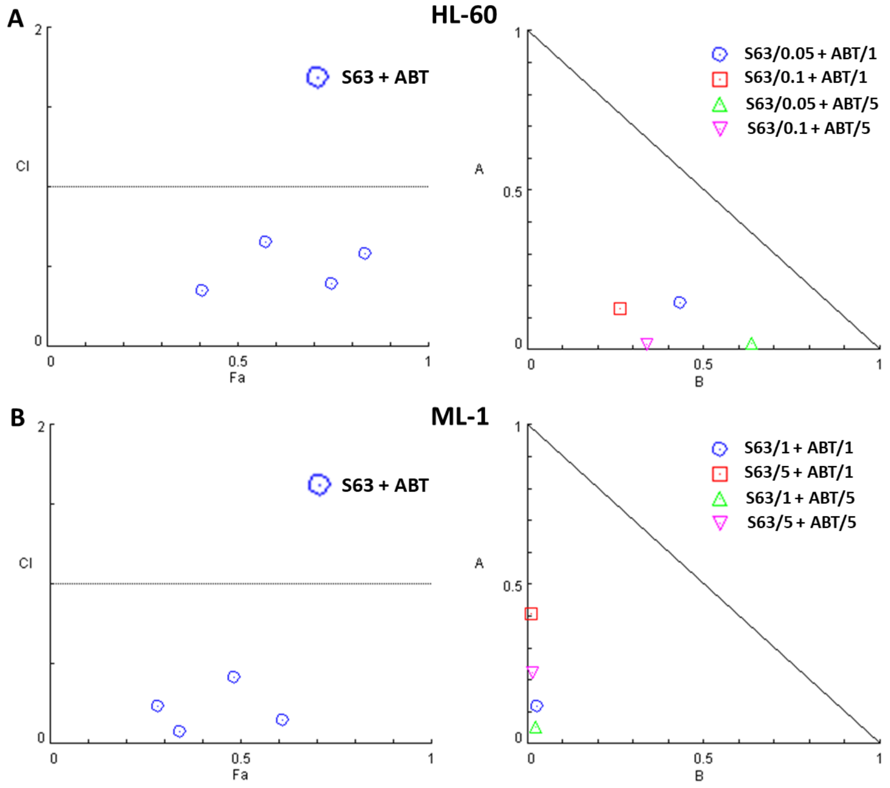


**Supplementary Figure S1.** The combination index (CI) values corresponding to the S63845 (S63) combinations with ABT-737 (ABT) were determined by isobologram analysis. A representative normalized isobologram and fraction-affected (Fa)-CI plots generated for HL-60 (A) and ML-1 (B) cells exposed to the action of S63845 and ABT-737 graphically depict the interaction between the tested agents. Points falling below the line indicate synergism. Designations 0.05, 0.1, 1 and 5 indicate the agent concentrations (µM).

*2.5. MAPK pathway inhibition potentiates the pro-apoptotic and pro-differentiating effects of S63845 in AML cells*

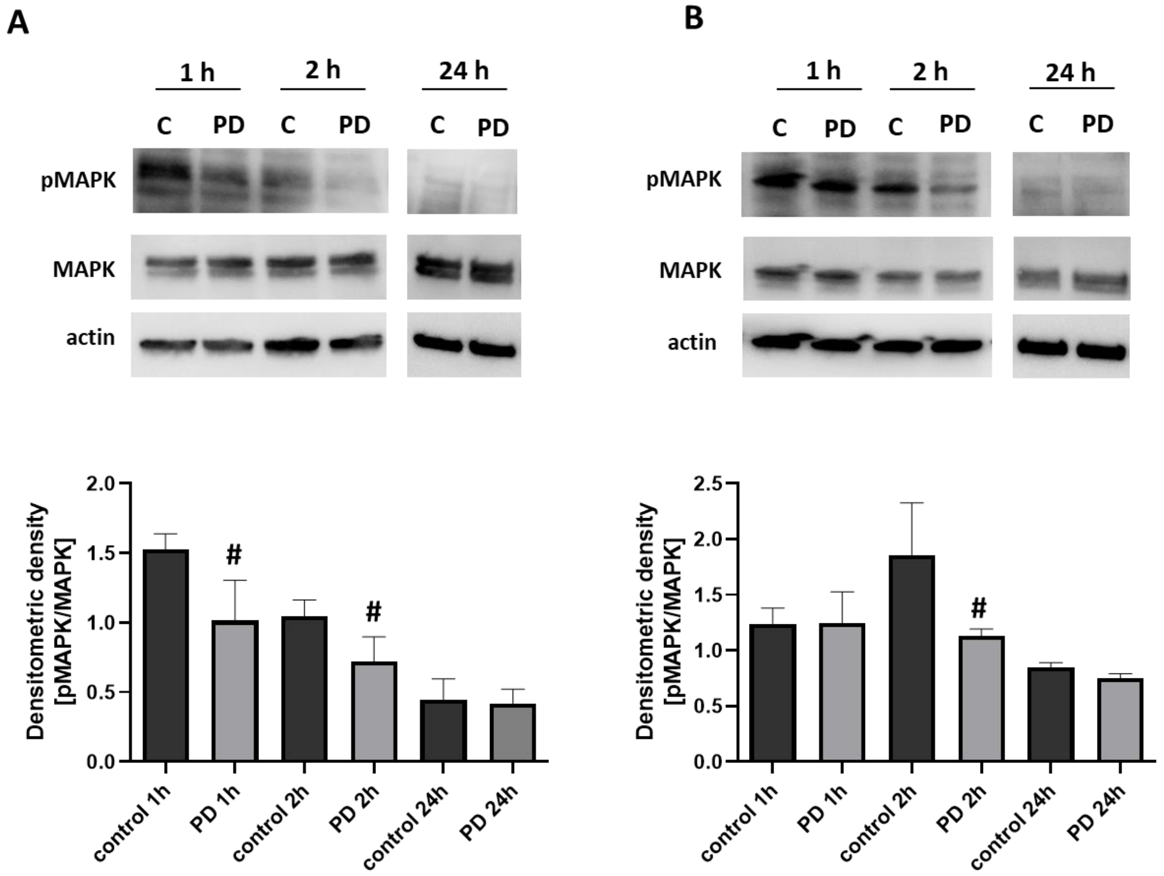


**Supplementary Figure S2.** Expression of phosphotylated and total MAPK protein in HL-60 (panel A) and ML-1 (panel B) cells after treatment with PD98059 (PD) for 1h, 2h and 24 h, detected by Western blotting. Relative levels of pMAPK and total MAPK were quantified by densitometry and normalized to β-actin. The graphs represent the ratio of pMAPK to total MAPK. Values are reported as the means ± SD of three independent experiments. Values significantly different ($p < 0.05$) according to one-way ANOVA are designated by # compared to the corresponding control.

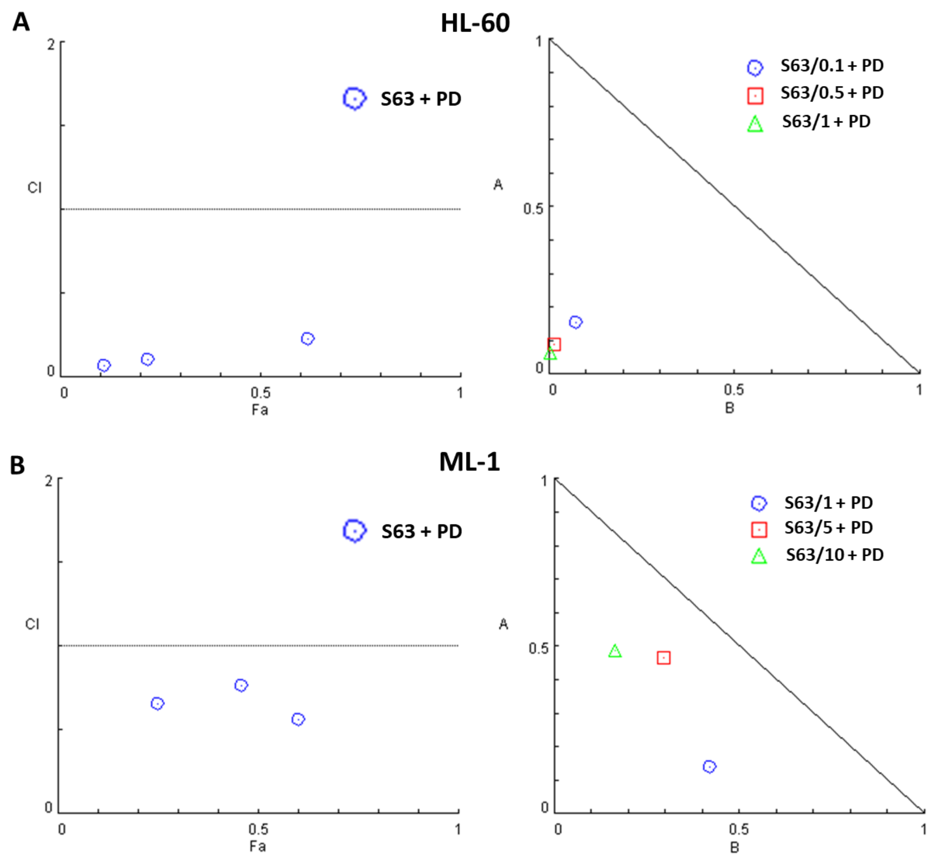


**Supplementary Figure S3.** The combination index (CI) values corresponding to the S63845 (S63) combinations with PD98059 (PD) were determined by isobologram analysis. A representative normalized isobologram and fraction-affected (Fa)-CI plots generated for HL-60 (A) and ML-1 (B) cells exposed to the action of S63845 and PD98059 graphically depict the interaction between the tested agents. Points falling below the line indicate synergism. Designations 0.1, 0.5, 1, 5 and 10 indicate the agent concentrations (μM).
